# Advancing Colorectal Cancer Screening: A Comprehensive Systematic Review of Artificial Intelligence (AI)-Assisted Versus Routine Colonoscopy

**DOI:** 10.7759/cureus.45278

**Published:** 2023-09-15

**Authors:** Jingle Thomas, Rakshana Ravichandran, Aiswarya Nag, Lovish Gupta, Mansi Singh, Binay K Panjiyar

**Affiliations:** 1 Internal Medicine, Al-Ameen Medical College, Vijayapura, IND; 2 Internal Medicine, Rajarajeswari Medical College and Hospital, Bangalore, IND; 3 Internal Medicine, Sri Ramachandra Institute of Higher Education and Research, Chennai, IND; 4 Internal Medicine, Maulana Azad Medical College, New Delhi, IND; 5 Medicine, O.O. Bogomolets National Medical University, Kyiv, UKR; 6 GCSRT, PGMEE, Harvard Medical School, Boston, USA; 7 Internal Medicine, California Institute of Behavioral Neurosciences & Psychology, Fairfield, USA

**Keywords:** polyp detection, adenoma detection, colorectal adenomas, colorectal polyps, screening, computer-aided detection, cade, artificial intelligence, colonoscopy, colorectal cancer

## Abstract

Colorectal cancer (CRC) is a rapidly escalating public health concern, which underlines the significance of its early detection and the need for the refinement of current screening methods. In this systematic review, we aimed to analyze the potential advantages and limitations of artificial intelligence (AI)-based computer-aided detection (CADe) systems as compared to routine colonoscopy. This review begins by shedding light on the global prevalence and mortality rates of CRC, highlighting the urgent need for effective screening techniques and early detection of this cancer type. It addresses the problems associated with undetected adenomas and polyps and the subsequent risk of interval CRC following colonoscopy. The incorporation of AI into diagnostics has been studied, specifically the use of CADe systems which are powered by deep learning. The review summarizes the findings from 13 randomized controlled trials (RCTs) (2019-2023), evaluating the impact of CADe on polyp and adenoma detection.

The findings from the studies consistently show that CADe is superior to conventional colonoscopy procedures in terms of adenoma detection rate (ADR) and polyp detection rate (PDR), particularly with regard to small and flat lesions which are easily overlooked. The review acknowledges certain limitations of the included studies, such as potential performance bias and geographic limitations. The review ultimately concludes that AI-assisted colonoscopy can reduce missed lesion rates and improve CRC diagnosis. Collaboration between experts and clinicians is key for successful implementation. In summary, this review analyzes recent RCTs on AI-assisted colonoscopy for polyp and adenoma detection. It describes the likely benefits, limitations, and future implications of AI in enhancing colonoscopy procedures and lowering the incidence of CRC. More double-blinded trials and studies among diverse populations from different countries must be conducted to substantiate and expand upon the findings of this review.

## Introduction and background

Colorectal cancer (CRC) is the third most common cancer and the cause of mortality among both male and female cancer patients in the United States. It is also the leading cause of death due to cancer among men under the age of 50 years. Early detection of CRC before it advances is associated with a significant success rate in treating the condition and CRC-related mortality in the US has fallen by 57% from 1970 to 2020. This decline is attributed to improved treatment as well as enhanced screening practices that identify precancerous changes in its early stage. The overall death rate witnessed an approximate 2% annual fall from 2012 to 2020. However, mortality among adults under the age of 55 years has been constantly on the rise since the mid-2000s [1]. More than 1.9 million new cases and 930,000 deaths from CRC were estimated to have occurred globally in 2020. It is expected to rise to about 3.2 million new cases per year (a 63% increase) along with 1.6 million deaths per year (a 73% increase) by 2040 [2]. The likelihood of developing CRC increases with age, with incidence rates increasing by 80-100% within each successive five-year age group until the age of 50 and then by 20-30% from ages 55-59 years and beyond [3].

Colonoscopy is the most commonly performed endoscopic procedure in the US and is widely recognized as the gold standard for CRC screening and surveillance [3]. In 2018, the American Cancer Society recommended that CRC screening should commence at the age of 45 years, as early screening is an effective means of improving CRC prognosis and further reducing the social burden associated with the condition [3]. A large cohort study had previously revealed that colonoscopy could lead to a 68% reduction in CRC-related mortality when compared to the absence of endoscopic screening [4]. Despite implementing early screening practices, the burden of undetected CRC continues to be a significant concern and is linked to the subsequent development of interval CRC following colonoscopy [5]. According to a recently published meta-analysis of over 15,000 tandem colonoscopies, the missed rates for adenomas were as high as 26% and advanced adenomas were around 9% [6].

The missed diagnosis rate is influenced by two major factors: blind spots and recognition failure. While the former can potentially be resolved by employing wide-angle ranges or remote attachments, overcoming recognition failure by endoscopists poses a more formidable challenge, especially for small and flat lesions, which are prone to be missed regardless of their level of experience​​​​ [6,7]. Every colonoscopy comprises roughly 50,000 frames, equivalent to a rate of around 25-30 frames per second and one polyp might be identifiable within just a few frames, illustrating the potential for challenges in polyp recognition [8].

The adenoma detection rate (ADR) reflects the percentage of colonoscopies in which one or more adenomatous colorectal lesions are found and is the primary quality indicator for colonoscopy. Every 1% rise in ADR will lead to a 3% reduction in the risk of interval colorectal cancer [9]. Additionally, the polyp detection rate (PDR), which measures the proportion of detected hyperplastic polyps in the colon, is another crucial quality indicator for colonoscopy. Significant efforts have been undertaken to enhance both ADR and PDR as they are associated with a reduced risk of post-colonoscopy CRC [6]. Another commonly accepted performance measure for colonoscopy is the adenoma miss rate (AMR). It is determined by performing two successive colonoscopies on the same patient and tallying the lesions missed during the initial inspection but subsequently detected during the follow-up examination [10].

The increase in colonoscopy requirements, driven by variables such as increased population-based screening, increase in the proportion of older demographics, and expanded colonoscopy indications, emphasizes the growing significance of improving colonoscopy procedures [11]. In recent years, the integration of cutting-edge technology has ushered in remarkable developments in the field of medical diagnostics. Artificial intelligence (AI) stands out as a transformative force among these technologies. Machine learning, a subset of AI, empowers machines to learn from the data provided. It improves its performance over time, enabling machines to analyze and interpret data in ways identical to human cognition.

Due to the significant advancement of AI, a remarkable innovation has emerged in the form of computer-aided detection (CADe) systems, which use deep learning, a type of machine learning method. The development of CADe has garnered substantial attention worldwide in the field of endoscopy, primarily due to its seamless integration into conventional endoscopy setups. CADe is reported to significantly improve the detection of colorectal neoplasia by promptly alerting endoscopists to suspected lesions in real time.

Several randomized controlled trials (RCTs) have been conducted and published in recent years on the effectiveness of CADe in detecting adenomas and polyps. In this systematic review, we aim to evaluate these studies regarding the potential benefits and limitations of CADe. The objective is to determine whether there is a significant difference in the detection rates of adenomas and polyps between AI-assisted colonoscopy and routine colonoscopy.

## Review

Methods

**Figure 1:**
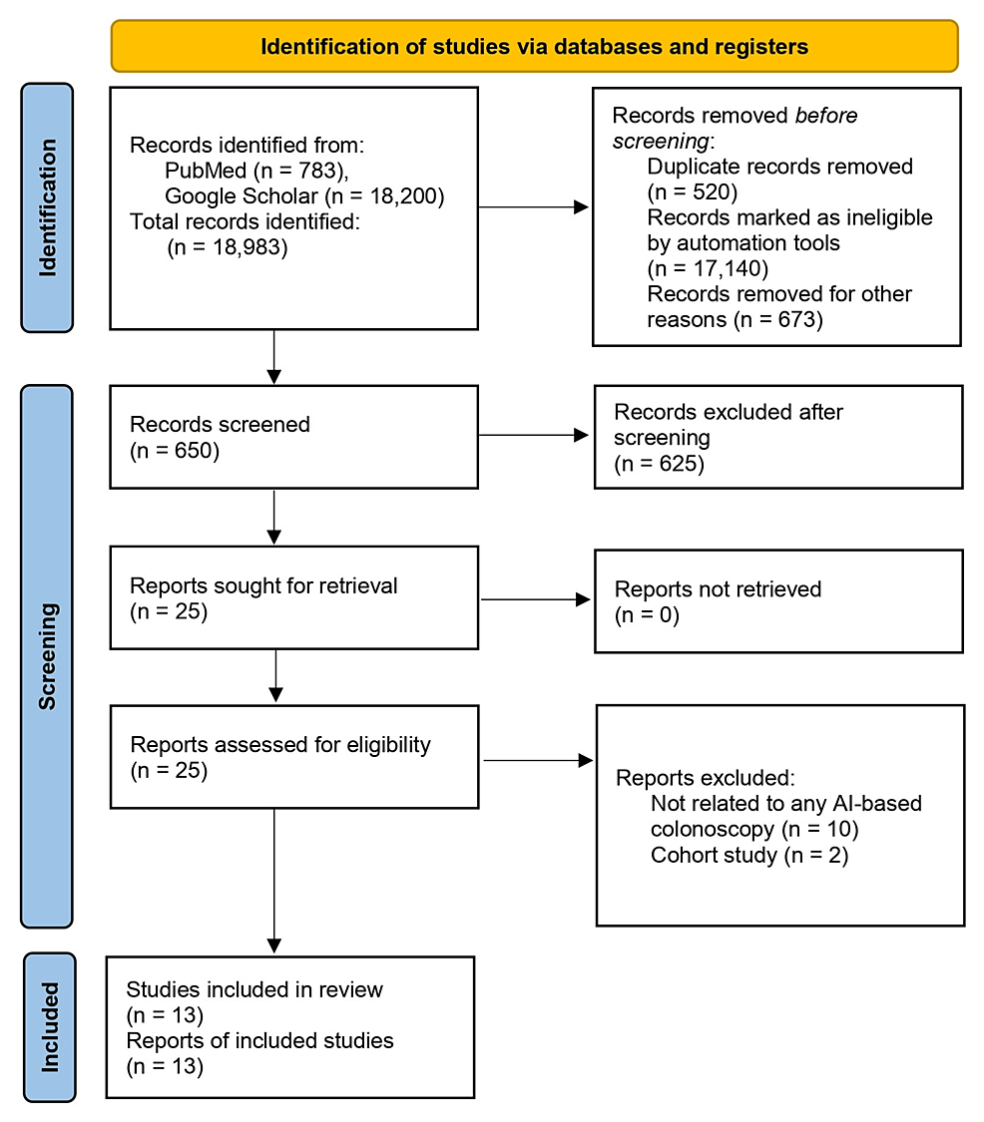
PRISMA flow diagram depicting the search strategy and study selection process PRISMA: Preferred Reporting Items for Systematic Reviews and Meta-Analyses

We developed the framework of the present systematic review based on the methodological framework recommended by the Cochrane Handbook [12]. This systematic review was conducted in adherence to the Preferred Reporting Items for Systematic Reviews and Meta-Analyses (PRISMA) guidelines 2020 [13]. The PRISMA flowchart Figure 1 depicts the entire study selection process. Since our data collection was confined to published papers, ethical approval was not required for this review.

Systematic Literature Search and Study Selection

We performed a comprehensive systematic literature search with no restrictions on PubMed (including Medline) and Google Scholar databases from their inception until July 2023.

Inclusion and Exclusion Criteria

We established specific criteria for including and excluding studies to achieve our objectives. Our Inclusion and exclusion criteria are listed in Table 1 and were established based on discussion among the authors to include quality studies.

**Table 1:** Inclusion and exclusion criteria

Inclusion criteria	Exclusion criteria
Population: all adults (aged >18 years) undergoing colonoscopy	Animal studies
Intervention: AI-assisted colonoscopy	Non-English language studies
Comparison: routine colonoscopy	Observational studies, correspondences or editorials, case reports, abstracts, and reviews
Outcome: adenoma detection rate (ADR), polyp detection rate (PDR)	Full text not available
Study design: only randomized controlled trials (RCTs) were included	

Only RCTs involving adult patients aged over 18 years that used an AI-powered system for the interventional group versus a standard group in whom a regular colonoscopy was employed were included in this review. Non-randomized studies, observational cohorts, case series, case reports, letters to editors, and other systematic reviews/meta-analyses were omitted. Also, articles in languages other than English and articles for which full text was not retrievable were excluded from this review.

Search Strategy

The search strings were applied using an ‘AND/OR’ framework by combining the following search terms using the Medical Subject Heading (MeSH) approach to construct search strategies: "Artificial intelligence, AI, Machine learning, Deep learning, Computer-aided diagnosis, GI, Gastrointestinal, Colonoscopy, Endoscopy, Colon examination, and Colorectal examination." The search strategies used in the databases are summarized in Table 2. To ensure that no relevant studies were omitted, we also performed a manual search of references of articles and reviews for additional potentially eligible studies.

**Table 2:** Search strategy and the number of results retrieved

Database	Search strategy	Results
PubMed	("Artificial intelligence" OR "AI" OR "Machine learning" OR "Deep learning" OR "Computer-aided diagnosis") AND ("GI" OR "Gastrointestinal") AND ("Colonoscopy" OR "Endoscopy" OR "Colon examination" OR "Colorectal examination")	783
Google Scholar	("Artificial intelligence" OR "AI" OR "Machine learning" OR "Deep learning" OR "Computer-aided diagnosis") AND ("GI" OR "Gastrointestinal") AND ("Colonoscopy" OR "Endoscopy" OR "GI endoscopy" OR "Colon examination" OR "Colorectal examination")	18,200

The retrieved studies from the databases were imported to Rayyan software; a check was performed to identify duplicate articles and all duplicate articles were removed after careful evaluation of the data. Four authors (JT, RR, AN, and LG) carried out the title and abstract screening, followed by a full-text screening of the selected articles. To ensure the quality of the review, each article was reviewed by two authors, which involved the abstract as well as full-text screening. Disagreements were resolved by reaching a consensus based on a discussion with a third author.

All authors extracted study data into Microsoft Excel. The data extracted were as follows: Sl. no., author, year, country, title, participant count, study design, AI technology used, and conclusions.

The bibliographic management tool utilized in this study was Mendeley Reference Manager.

Quality Appraisal

**Figure 2:**
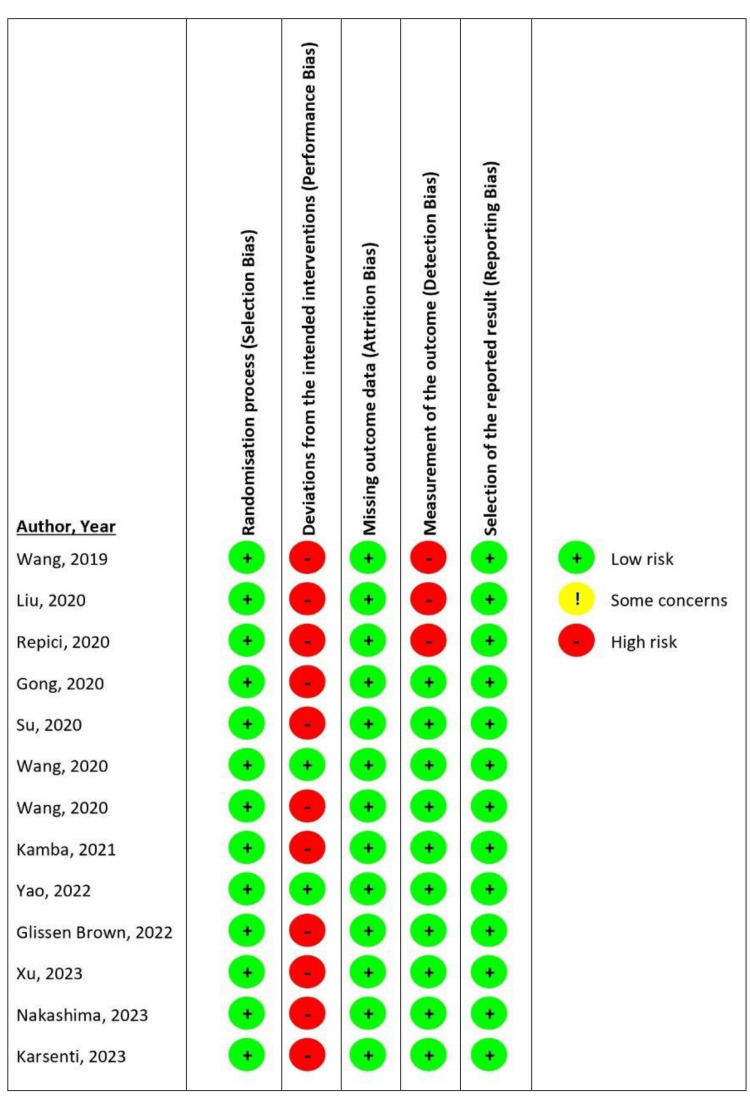
Summary of the application of the Cochrane risk of bias tool for randomized trials RoB 2: Risk of bias 2 Studies - Wang et al., 2019 [14], Liu et al., 2020 [15], Repici et al., 2020 [8], Gong et al., 2020 [16], Su et al., 2020 [17], Wang et al., 2020 [18], Wang et al., 2020 [19], Kamba et al., 2021 [20], Yao et al., 2022 [21], Glissen Brown et al., 2022 [22], Xu et al., 2023 [23], Nakashima et al., 2023 [24], Karsenti et al., 2023 [25]

The Cochrane tool was used to assess for risk of bias in the included RCTs. Two authors (JT, RR) independently assessed each RCT for the risk of bias. The risk in each study was rated as high, low, or having some concerns with respect to each of the five domains of the tool: Randomization process (selection bias), deviations from the intended interventions (performance bias), missing outcome data (attrition bias), measurement of the outcome (detection bias), and selection of the reported result (reporting bias). Consensus was reached through discussion, and when needed, disagreements were resolved by discussing with the other authors. Figure 2 depicts a summary of the application of the Cochrane risk of bias tool for randomized trials (RoB 2) for each of the included RCTs.

Results

The database search identified 18,983 potential articles including 783 from PubMed/MEDLINE and 18,200 from Google Scholar databases. Each paper was carefully reviewed and filtered based on the inclusion and exclusion criteria, which led to the exclusion of 18,333 articles. The remaining 650 papers were closely examined and 637 more were excluded as their content did not meet the inclusion criteria after a thorough screening process. Finally, a quality check was done on the remaining 13 papers, which met all the criteria of the quality appraisal tool.

All 13 studies included in the final analysis were RCTs, and each of these studies strictly adhered to defined protocols, ensuring the rigor of their methodology. A total of 12,693 participants were involved in the trials. The trials were published from 2019 to 2023. Among the 13 studies, eight were conducted in China, two in Japan, one in France, one in Italy, and one in the USA; three out of 13 trials were non-blinded [8,14,15], eight trials were single-blinded RCTs [16,17,19,20,22-25], and two were double-blinded RCTs [18,21]. Table 3 provides a detailed description of each study.

**Table 3:** Summary of the included studies ADR: adenoma detection rate; CADe: computer-aided detection; PDR: polyp detection rate; AQCS: automatic quality control system; DCNN: deep convolutional neural network; CAQ: computer-aided quality improvement

Sl. no.	Study	Country	Title	Participant count	Study design	AI technology used	Conclusions
1.	Wang et al., 2019 [14]	China	Real-time automatic detection system increases colonoscopic polyp and adenoma detection rates: a prospective randomized controlled study	1058	Non-blinded, prospective randomized controlled trial	A real-time automatic polyp detection system (Shanghai Wision AI Co., Ltd.)	In a population with a low occurrence of ADR, utilizing an automated polyp detection system during colonoscopy led to a significant rise in the identification of small adenomas and hyperplastic polyps
2.	Liu et al., 2020 [15]	China	Study on detection rate of polyps and adenomas in artificial-intelligence-aided colonoscopy	1026	Non-blinded, prospective randomized controlled trial	Deep‑learning‑based CADe system	The CADe system is feasible for enhancing the identification of polyps and small adenomas during colonoscopy procedures
3.	Repici et al., 2020 [8]	Italy	Efficacy of real-time computer-aided detection of colorectal neoplasia in a randomized trial	685	Non-blinded, prospective randomized controlled trial	AI-powered medical device (GI Genius, Medtronic)	The CADe group experienced a significant increase in ADR compared to the control group, especially smaller to medium-sized ones, showcasing the safety and effectiveness of integrating CADe into real-time colonoscopy
4.	Gong et al., 2020 [16]	China	Detection of colorectal adenomas with a real-time computer-aided system (ENDOANGEL): a randomized controlled study	704	Single-blind, prospective randomized controlled trial	ENDOANGEL - real-time quality improvement system based on deep learning	The ENDOANGEL system significantly improved the ADR and PDR during colonoscopy and appears to be safe and effective for use during routine colonoscopy
5.	Su et al., 2020 [17]	China	Impact of a real-time automatic quality control system on colorectal polyp and adenoma detection: a prospective randomized controlled study (with videos)	659	Single-blind, prospective randomized controlled trial	Automatic Quality Control System (AQCS) that incorporates Deep Convolutional Neural Network (DCNN) models	The study revealed notable enhancements in the AQCS-assisted group, including higher rates of ADR and PDR, as compared to the control group
6.	Wang et al., 2020 [18]	China	Effect of a deep learning computer-aided detection system on adenoma detection during colonoscopy (CADe-DB trial): a double-blind randomized study	1046	Double-blind, prospective randomized controlled trial	CADe system (EndoScreener; Wision AI, Shanghai, China)	The polyps and adenomas initially missed by the endoscopists could be detected using a high-performance CADe system during colonoscopy
7.	Wang et al., 2020 [19]	China	Lower adenoma miss rate of computer-aided detection-assisted colonoscopy versus routine white-light colonoscopy in a prospective tandem study	369	Single-blind, prospective randomized controlled trial	CADe system (EndoScreener)	The employment of CADe systems during colonoscopy notably decreased the adenoma and polyp miss rates when compared to routine colonoscopy
8.	Kamba et al., 2021 [20]	Japan	Reducing adenoma miss rate of colonoscopy assisted by artificial intelligence: a multicenter randomized controlled trial	358	Single-blind, prospective randomized controlled trial	CADe system (LPIXEL Inc.)	Utilizing CADe systems led to a decrease in the rate of missed adenomatous lesions during colonoscopy procedures with an increased adenoma detection rate
9.	Yao et al., 2022 [21]	China	Effect of an artificial intelligence-based quality improvement system on the efficacy of a computer-aided detection system in colonoscopy: a four-group parallel study	1076	Double-blind, prospective randomized controlled trial	ENDOANGEL - real-time quality improvement system based on deep learning	Integrating both CADe and CAQ systems increased the ADR and significantly enhanced the effectiveness of CADe
10.	Glissen Brown et al., 2022 [22]	USA	Deep learning computer-aided polyp detection reduces adenoma miss rate: a United States multi-center randomized tandem colonoscopy study (CADeT-CS Trial)	223	Single-blind, prospective randomized controlled trial	CADe system (EndoScreener)	CADe can diminish variations among providers in the quality of colonoscopy by decreasing the rate of missed adenomas, even among experienced practitioners
11.	Xu et al., 2023 [23]	China	Artificial intelligence–assisted colonoscopy for colorectal cancer screening: a multicenter randomized controlled trial	3059	Single-blind, prospective randomized controlled trial	AI polyp detection system (Eagle-Eye)	AI-assisted colonoscopy improved overall ADR, advanced ADR, and ADR of both expert and nonexpert attending endoscopists
12.	Nakashima et al., 2023 [24]	Japan	Clinical evaluation of computer-aided colorectal neoplasia detection using a novel endoscopic artificial intelligence: a single-center randomized controlled trial	415	Single-blind, prospective randomized controlled trial	Novel CADe system, namely, CAD EYE	The utilization of the CADe system during colonoscopy resulted in a superior ADR compared to procedures conducted solely by skilled endoscopists
13.	Karsenti et al., 2023 [25]	France	Effect of real-time computer-aided detection of colorectal adenoma in routine colonoscopy (COLO-GENIUS): a single-center randomized controlled trial	2015	Single-blind, prospective randomized controlled trial	CADe-assisted colonoscopy (GI Genius 2.0.2; Medtronic)	The findings support the benefits of CADe, even in a non-academic center and the systematic use of CADe in routine colonoscopy is to be considered

Summary of Studies

In 2019, Wang and colleagues [14] conducted a research study delving into the impact of incorporating deep learning into an automated polyp detection system. They aimed to see how it affected the ADR and PDR during colonoscopies, by conducting an open, non-blinded trial. Their trial included 1058 participants and the results showed that the CADe group had a significantly higher ADR (29.1%) compared to the control group (20.3%) (p<0.001).

The following year, Liu et al. [15] also took a closer look at the CADe system's influence on ADR and PDR during colonoscopies. They randomly assigned 1026 patients to receive the procedure with or without CADe assistance. The study revealed that the CADe group had a 5.8% increase in adenoma detection compared to the standard group. This method also enhanced the detection of small adenomas and proliferative polyps with a statistically significant difference (p<0.001).

In 2020, Repici et al. [8] investigated the effectiveness and safety of a CADe system for identifying colorectal neoplasias. They enrolled 685 patients and found that the CADe group had a significantly higher ADR (54.8%) compared to the control group (40.4%). This approach improved the detection of adenomas per colonoscopy, especially small and medium-sized adenomas.

Gong et al. [16], also in 2020, studied an AI system called ENDOANGEL, which employs deep neural networks and algorithms to improve adenoma detection during colonoscopies. With 704 participants, the ENDOANGEL-assisted group exhibited a notably higher ADR (16%) compared to the control group (8%) (p=0.0010). PDR was also higher (47%) than the control group (34%) (p=0.0016).

Su et al. [17] developed an automated quality control system (AQCS) in 2020, aiming to improve routine colonoscopy effectiveness. In a trial involving 659 patients, the AQCS-assisted group showed a substantially higher ADR (28.9%) compared to the control group (16.5%) (p<0.001). PDR was also higher (38.3%) than the control group (25.4%) (p=0.001), demonstrating the effectiveness of AQCS.

In the same year, Wang et al. [18] conducted a double-blind randomized trial involving 962 participants to assess the CADe system's efficiency in identifying colon polyps and adenomas during colonoscopies. The CADe group displayed a significantly higher ADR (34%) compared to the control group (28%) (p=0·030). PDR was also higher (52%) than the control group (37%) (p<0·0001).

In a study by Wang et al. (2020) [19], the researchers analyzed how well CADe systems could find precancerous polyps during colonoscopies. They split participants into CADe and routine groups, focusing on the adenoma miss rate (AMR) and polyp miss rate (PMR). Among 369 participants, the CADe group had a much lower AMR (13.89%) than the control group (40%) (p<0.0001). PMR was also lower (12.98%) compared to the control group (45.9%) (p=0.0001). This showed that using CADe during colonoscopies significantly cut down on missing adenomas and polyps, hinting at CADe's potential to detect overlooked adenomas effectively and decrease the incidence of interval CRC.

Kamba et al. (2021) [20] looked into whether using a CADe system could make adenoma detection more accurate during colonoscopy screenings in Japan. They classified patients randomly into a standard colonoscopy group and a CADe-first group, having them undergo a tandem procedure. Among 358 participants, the CADe group had a significantly lower AMR (13.8%) compared to the control group (36.7%) (p<0.0001). PMR was also lower (14.2%) than the control group (40.6%) (p=0.0001). Moreover, using CADe improved the ADR (64.5%) compared to standard colonoscopy (53.6%; p=0.036).

In a 2022 study by Yao et al. [21], the efficacy of combining a CADe system with a computer-aided quality improvement (CAQ) system to enhance the ADR was investigated. The study involved 1076 patients who were assigned randomly into four different treatment groups. The ADRs were 14.76%, 21.27%, 24.54%, and 30.60% in the control, CADe, CAQ, and COMBO groups, respectively. In comparison to the control group, both the CADe and CAQ groups exhibited notably higher ADRs. The findings indicated that the group utilizing both CADe and CAQ systems displayed a notably higher ADR compared to the group using CADe alone. The study concluded that integrating both CADe and CAQ systems significantly enhanced the effectiveness of CADe.

In a randomized study by Glissen Brown et al. (2022) [22], the efficacy of CADe systems in identifying colon polyps and adenomas during colonoscopy and minimizing the rate of missed adenomas was examined. Patients presenting for CRC screening were randomized to CADe colonoscopy first or high-definition white light (HDWL) colonoscopy first, followed immediately by the other procedure in tandem fashion by the same endoscopist. The study involved 223 participants, with the CADe group showing a significantly lower AMR (20.12%) compared to the control group (31.25%) (p=0.0247). PMR was also lower (20.7%) than the control group (33.71%) (p=0.0007).

In 2023, Xu et al. [23] conducted a single-blind RCT that aimed to assess the efficacy of AI-assisted colonoscopy in an asymptomatic population eligible for CRC screening and involved endoscopists with varying levels of experience. A total of 3059 patients underwent colonoscopies, randomly assigned to receive the procedure either with or without the assistance of the AI-assisted colonoscopy. The CADe group showed a substantially higher ADR (39.9%) compared to the control group (32.4%) (p<0.001). The outcomes showed that AI-assisted colonoscopy improved overall ADR, advanced ADR, and ADR of both expert and nonexpert attending endoscopists.

In 2023, Nakashima et al. [24] also conducted a single-center, single-blind prospective randomized study in Japan to assess the clinical performance of CADe to detect colonic adenomas compared to the control group. A total of 415 patients underwent colonoscopies, randomly assigned to either the CADe group or the control group. The colonoscopies performed with CADe had a higher ADR (39.9%) compared to the control group (32.4%) (p<0.001). The AMRs were 11.9% in the CADe group and 26.0% in the control group (p=0.037).

A single-center randomized controlled trial study on the efficacy of a CADe system in polyp detection during colonoscopy was conducted by Karsenti et al. [25] in 2023. A total of 2015 patients were randomly assigned to either standard colonoscopy or CADe-assisted colonoscopy. The CADe group showed a higher ADR (37·5%) compared to the control group (33·7%) (p=0·051). PDR was also higher (45%) than the control group (41%) (p=0·048).

Discussion

CRC is a significant public health concern due to its high mortality rate and high prevalence [1]. With missed rates for adenomas as high as 26% (9% for advanced adenomas), the underlying cause becomes clear: nearly 85% of these interval cancers can be attributed to either undetected polyps or incomplete polyp removal during prior procedures [6,26]. Routine colonoscopy has been used as the standard procedure to diagnose and prevent GI lesions for a long time, resulting in early intervention and better patient outcomes. However, recent breakthroughs in AI have led to a major change in how we approach lesion identification. The goal of this review was to provide a comprehensive analysis of AI-assisted detection and routine colonoscopy for detecting GI lesions, shedding light on the possible benefits of both.

Our systematic review included a total of 13 RCTs conducted from 2019 to 2023. The focus of these trials was on the use of AI-assisted colonoscopy for detecting colorectal polyps and adenomas. The studies were diverse in terms of their location (China, Japan, France, Italy, USA), blinding status (non-blinded, single-blinded, double-blinded), and sample size. Overall, the studies aimed to evaluate the impact of AI-assisted systems, particularly CADe, on the detection rates of polyps and adenomas during colonoscopy.

Across all RCTs included in the review, ADR emerged as the most significant and essential quality measure. These studies uniformly recognized that ADR plays a critical role in assessing colonoscopy quality as it directly correlates with the number of colorectal adenomas found during the procedure. The other quality indicators used to measure the efficacy of CADe compared to routine colonoscopies are PDR and AMR. PDR measures the overall capability to detect various polyps, while AMR evaluates the rate at which initially overlooked adenomas are identified upon subsequent examinations. Additionally, PMR was also examined, shedding light on AI's impact on the thoroughness of polyp detection during colonoscopy.

Several consistent trends emerged from the reviewed studies. The utilization of CADe systems led to improvements in the ADR and the PDR compared to conventional colonoscopy. Studies reported statistically significant differences in the quality indicators among the AI-assisted and control groups. The incorporation of CADe systems resulted in higher ADR, indicating the potential for improved identification of adenomas. The findings also highlighted the effectiveness of CADe systems in detecting small adenomas and hyperplastic polyps, which are often challenging to identify.

All the included trials in this study demonstrated better rates of detecting lesions, polyps, adenomas, and mean polyps per procedure. However, in actual practice, the rate of lesion detection may be less than what is reported since ADRs and PDRs from routine colonoscopies in the trials may be overrepresented in single-blinded studies due to the Hawthorne effect, as the observation of operating endoscopists might have improved lesion detection as they become aware of the study being documented. Previous studies [27,28] have reported that adding a second observer to colonoscopies led to significantly increased ADRs. These AI-augmented CADe systems effectively counteract the possibility of fatigued endoscopists missing polyps, acting as a diligent second observer that mitigates the risk of errors arising from extended procedures. Additionally, AI's guidance proves invaluable for less experienced endoscopists, reducing errors and accelerating their learning curve.

Eight of the 13 RCTs included in this review were conducted in a single country (China, which has a lower risk of CRC and ADR than Western countries [29]). Hence, the findings of these trials may not be generalizable to other countries with higher baseline ADR and PDR. Three of the included trials were non-blinded and eight were single-blinded, and hence they may have been susceptible to performance bias. The findings of these studies may reflect an underestimation or overestimation of AI-assisted colonoscopy. Only two studies [18,21] were designed as double-blinded RCTs, and only one of them was a truly double-blinded RCT. Wang et al. used a sham system to keep both the patients and the endoscopist unaware of the random assignment, and in the other study [21], while the endoscopy operators were informed about the allocation of the patient by the researchers, patients and outcome assessors were blinded to intervention allocation.

The results of these studies have significant implications for clinical practice. AI-assisted colonoscopy, particularly through CADe systems, holds promise for enhancing the accuracy of adenoma and polyp detection. The integration of AI technology into routine colonoscopy procedures has the potential to reduce the rates of missed lesions and improve overall CRC diagnosis and prevention. Future directions for research include designing more double-blinded RCTs to decrease the performance bias. Further trials where the screening populations involve a more diverse group of patients will help better define the effectiveness of the AI systems. Trials should be conducted in many other countries apart from China to assess the generalizability of these findings.

The strengths of this review are as follows: a thorough systematic literature search with well-defined inclusion and exclusion criteria, and a rigorous evaluation of the risk of bias using the Cochrane tool. We also included more studies than previous systematic reviews, involving more geographic locations, and tried to enhance the generalizability of the study. Additionally, only RCTs were included in this review, with a view to improving the reliability of our estimates in terms of the real-time use of AI.

We must also acknowledge that our systematic review has a few limitations. We limited our analysis to articles published in the English language and strictly adhered to the inclusion and exclusion criteria to include the relevant studies. One limitation of CADe itself is that if a mucosal segment is not visualized on the screen, the technology may not contribute to an improvement in ADR. Therefore, it is necessary to conduct studies that encompass varying levels of bowel preparation (poor, adequate, and excellent) to make the findings more broadly applicable. In addition to enhancing lesion and polyp detection, the use of CADe systems in colonoscopy may also slightly extend procedure times. However, this increase in time is often outweighed by improved lesion detection. The actual impact on procedure time can vary based on factors like the endoscopist's familiarity with CADe, case complexity, and the specific AI technology used. Future research should comprehensively assess the effect on procedure time while considering these variables to better understand its efficiency in colonoscopy.

While the included studies primarily focused on CADe detection rates, there was limited data on the missed rates associated with CADe. Future studies should assess the potential impact of missed rates of CADe and the psychological implications it may have with regard to endoscopists second-guessing their findings when CADe fails to detect lesions. Another potential limitation is that the endoscopists in our included studies were experienced gastroenterologists, which might have led to the underestimation of the true impact of AI-assisted colonoscopy in settings where endoscopists have varying levels of experience.

Another limitation is the lack of data in terms of categorizing the effectiveness of CADe based on factors like gender and race, as well as its adoption across various socioeconomic groups. Furthermore, evaluating the cost-effectiveness of AI-assisted colonoscopy in comparison to conventional colonoscopy requires more validation and extensive evidence to reach substantial conclusions. Conducting more studies on the expenses linked to CADe can have implications for its feasibility, accessibility, and utility, particularly among individuals in lower socioeconomic classes.

## Conclusions

This systematic review provided a comprehensive overview of recent RCTs investigating the efficacy of AI-assisted colonoscopy for the detection of colorectal polyps and adenomas. The reviewed studies consistently demonstrated the positive impact of AI-assisted systems, particularly CADe, on improving adenoma and polyp detection rates. These findings highlight the potential for AI technology to play a transformative role in enhancing the effectiveness of colonoscopy procedures and subsequently reducing the burden of CRC. Further research and clinical implementation are warranted to harness the full potential of AI-assisted CADe in routine clinical settings. Also, collaborative efforts between AI experts, endoscopists, and clinicians are imperative for its successful implementation.
